# Trends in Mortality Due to Cardiovascular Diseases Among Patients With Parkinson's Disease in the United States: A Retrospective Analysis

**DOI:** 10.1002/clc.70079

**Published:** 2025-01-16

**Authors:** Muzamil Akhtar, Hanzala Ahmed Farooqi, Rayyan Nabi, Sabahat Ul Ain Munir Abbasi, Sarah MacKenzie Picker, Raheel Ahmed

**Affiliations:** ^1^ Gujranwala Medical College Gujranwala Pakistan; ^2^ Islamic International Medical College Riphah International University Islamabad Pakistan; ^3^ Allama Iqbal Medical College Lahore Pakistan; ^4^ Sunderland Royal Hospital Sunderland UK; ^5^ National Heart and Lung Institute, Imperial College London London UK

**Keywords:** cardiovascular diseases, CDC WONDER, mortality trends, Parkinson's disease

## Abstract

**Background:**

Parkinson disease (PD) and cardiovascular diseases (CVD) present significant health burdens, particularly among older adults. Patients with PD have an elevated risk of CVD‐related mortality. Analyzing mortality trends in this population may help guide focused interventions.

**Methods:**

Mortality data were extracted from the CDC WONDER database, using ICD‐10 code G20 for PD and I00‐I99 for CVD. Age‐adjusted mortality rates (AAMR) per 100,000 were calculated and trends were examined across variables including gender, year, race, and urbanization, place of death, region, and state. Annual percentage change (APC) with 95% confidence intervals (CI) was computed using Joinpoint regression.

**Results:**

A total of 138 151 CVD‐related deaths were reported among individuals with PD. The AAMR decreased from 23.5 in 1999 to 12.7 in 2020, with a notable decline between 1999 and 2014 (APC: −5.13; 95% CI, −5.44 to −4.86), followed by a modest increase from 2014 to 2020 (APC: 1.37; 95% CI, 0.16–3.05). Males exhibited higher AAMRs compared to females (Male AAMR: 22.6 vs. Female AAMR: 10.4). Non‐Hispanic (NH) Whites had the highest AAMR (16.1), followed by Hispanics (11.2), NH Asians (10.2), and NH Blacks (9.7). Nonmetropolitan areas showed a higher AAMR (16.3) compared to metropolitan areas (14.9). State‐level analysis indicated Nebraska with the highest AAMR (21.4), while Georgia recorded the lowest (9.9).

**Conclusions:**

CVD‐related mortality in PD patients has declined overall, though rates rose slightly from 2014 to 2020. Gender, racial, and geographic disparities highlight the need for targeted strategies to reduce cardiovascular risks in this population.

## Graphical abstract

Trends in cardiovascular mortality among Parkinson's disease patients from 1999 to 2020 reveal a decline in AAMR, with disparities across gender, race, and geography. Men and non‐Hispanic Whites show higher AAMRs. Nonmetropolitan areas and the West report elevated rates. Overall, modest increases are noted post‐2014, highlighting demographic and regional differences in disease burden.

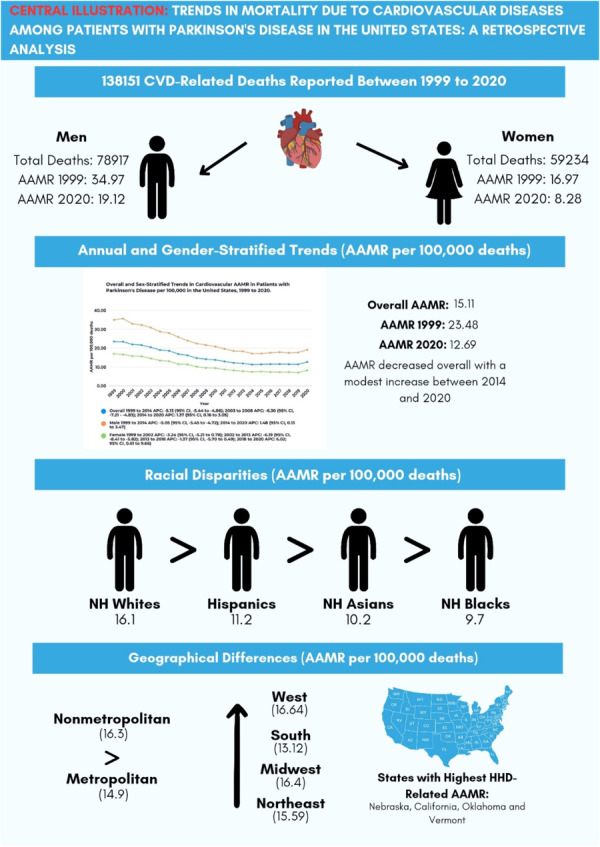

## 1 Introduction

Parkinson disease (PD) is an age‐related neurodegenerative disorder, for which no established preventive measures currently exist. As the population in the western world mostly comprises of older people, the incidence of neurodegenerative disorders like PD has increased, posing an estimated economic burden of $52B per year in the US [1]. Additionally, Arnold et al. found that the incidence of a new cardiovascular disease (CVD) in older Americans is high [2]. Therefore, older individuals are predisposed to the risk of developing both CVD and PD. It is important to understand that CVD and PD both share biological processes, particularly inflammation, insulin resistance, lipid metabolism, and oxidative stress [3].

The increased prevalence of CVD in PD patients arises from a complex interplay of autonomic dysfunction, medication effects, and genetic factors. Autonomic nervous system dysfunction, leading to cardiac dysautonomia, is the most common cardiovascular issue in PD, characterized by orthostatic and postprandial hypotension as well as supine and postural hypertension [4, 5, 6]. Medications, particularly levodopa—the mainstay treatment for PD—contribute to the prevalence of cardiovascular issues in PD patients [7]. While levodopa greatly enhances survival and quality of life, it has been linked to increased aortic stiffness, hypertension, and left ventricular diastolic dysfunction. Additionally, it can cause orthostatic hypotension, likely through a negative inotropic effect, although the exact mechanism remains unclear [8, 9]. Donepezil, an anticholinergic agent, has been associated with prolonged QT intervals during extended use [10].

Genes associated with PD, including α‐synuclein, Parkin, PINK1, DJ‐1, and LRRK2, may contribute to the connection between PD and coronary artery disease (CAD). These genes are expressed in heart tissue and are thought to play roles in oxidative stress and diminished cardioprotective mechanisms, particularly in cases of gene mutations or depletions [11, 12, 13]. These multifaceted mechanisms underscore the need for comprehensive cardiovascular evaluation and management in patients with PD to mitigate the heightened risk of CVD.

Given the high incidence along with the significant economic burden that they impose, analyzing mortality trends due to CVD among patients with PD is essential to better understand the relationship between these two diseases. Therefore, we conducted a retrospective analysis of data from the CDC Wonder (Centers for Disease Control and Prevention Wide‐Ranging Online Data for Epidemiologic Research) database to help illuminate the incidence and impact of CVD in patients with PD across various demographics and regions within the US, helping to identify and stratify at‐risk populations with PD who may require heightened monitoring. Such revelations can alert physicians regarding the prognosis of their patient, urging them to tailor their treatment strategies more effectively, ultimately enhancing patient outcomes.

## 2 Methods

### 2.1 Study Settings and Population

In this retrospective analysis, we initially extracted all data from the CDC Wonder database. The data retrieved were evaluated to understand the trends in mortality due to CVD among patients with PD in the US, ranging from 1999 to 2020. To help us obtain the relevant information we needed for our study, we used the following International Statistical Classification of Diseases and Related Health Problems‐10th Revision (ICD‐10) codes: G20 and I00‐I99 for PD and CVD, respectively. These codes have previously been used in literature to identify records of mortality due to PD and CVD [14, 15]. Death certificates listing PD as a multiple cause of death and CVD as the underlying cause of death in patients aged 65 years and older were analyzed. This study was exempt from local institutional review board approval because it used deidentified government‐issued public use data set and follows the STROBE (Strengthening the Reporting of Observational Studies in Epidemiology) guidelines for reporting.

### 2.2 Data Abstraction

Data was extracted for the following variables: year, gender, race, urbanization, place of death and state. Different races compared with each other included non‐Hispanic (NH) White, NH Black or African American, Hispanic or Latino, NH American Indian or Alaskan Native, and NH Asian or Pacific Islander. However, data for American Indians was not analyzed due to unreliable data. Location of death included medical facilities (outpatient, emergency room, inpatient, death on arrival, or status unknown), home, hospice, and nursing home/long‐term care facility. The National Center for Health Statistics Urban‐Rural Classification Scheme was used to assess the population by urban (large metropolitan area [population $1 million], medium/small metropolitan area [population 50 000–999 999] and rural [population < 50 000]) counties as per the 2012 US consensus [16]. Regions were stratified into Northeast, Midwest, South, and West according to the U.S. Census Bureau definitions [17].

### 2.3 Statistical Analysis

We calculated the crude and age‐adjusted mortality rates (AAMRs) per 100 000 population from 1999 to 2020 by year, gender, race/ethnicity, state, location of death and urban‐rural status with 95% CIs. This enabled us to effectively analyses mortality trends due to PD and CVD from 1999 to 2020 at a national level. The number of PD and CVD related deaths were divided by the corresponding U.S. population of that year to calculate crude mortality rates. AAMRs were calculated by standardizing PD and CVD‐related deaths to the year 2000 U.S. population [17]. Annual percent change (APC) values with 95% CI in AAMR were then calculated using the Joint point Regression Program (Join point V 4.9.0.0, National Cancer Institute). Not only did this allow us to quantify national trends, but also adjusted log‐linear regression models wherever temporal variations occurred. This method allows for the identification of significant changes in AAMR over time. Using 2‐tailed t‐testing, APCs were deemed to be increasing or decreasing if the slope characterizing the change in mortality was significantly different from zero. *p* < 0.05 was taken to be statistically significant.

## 3 Results

Between 1999 and 2020, a total of 138 151 death certificates were identified for individuals aged 65 years and older, listing cardiovascular disease (CVD) as the primary cause of death and Parkinson's disease (PD) as a contributory cause. Of these decedents, 57.1% (*n* = 78 917) were males and 42.9% (*n* = 59 234) were females. The majority of deaths occurred in nursing homes or long‐term care facilities (43.32%), followed by medical facilities (25.79%) and at the decedent's home (23.74%) (Supporting Information S1: Table 1).

## 4 Annual Trends

**Figure 1:**
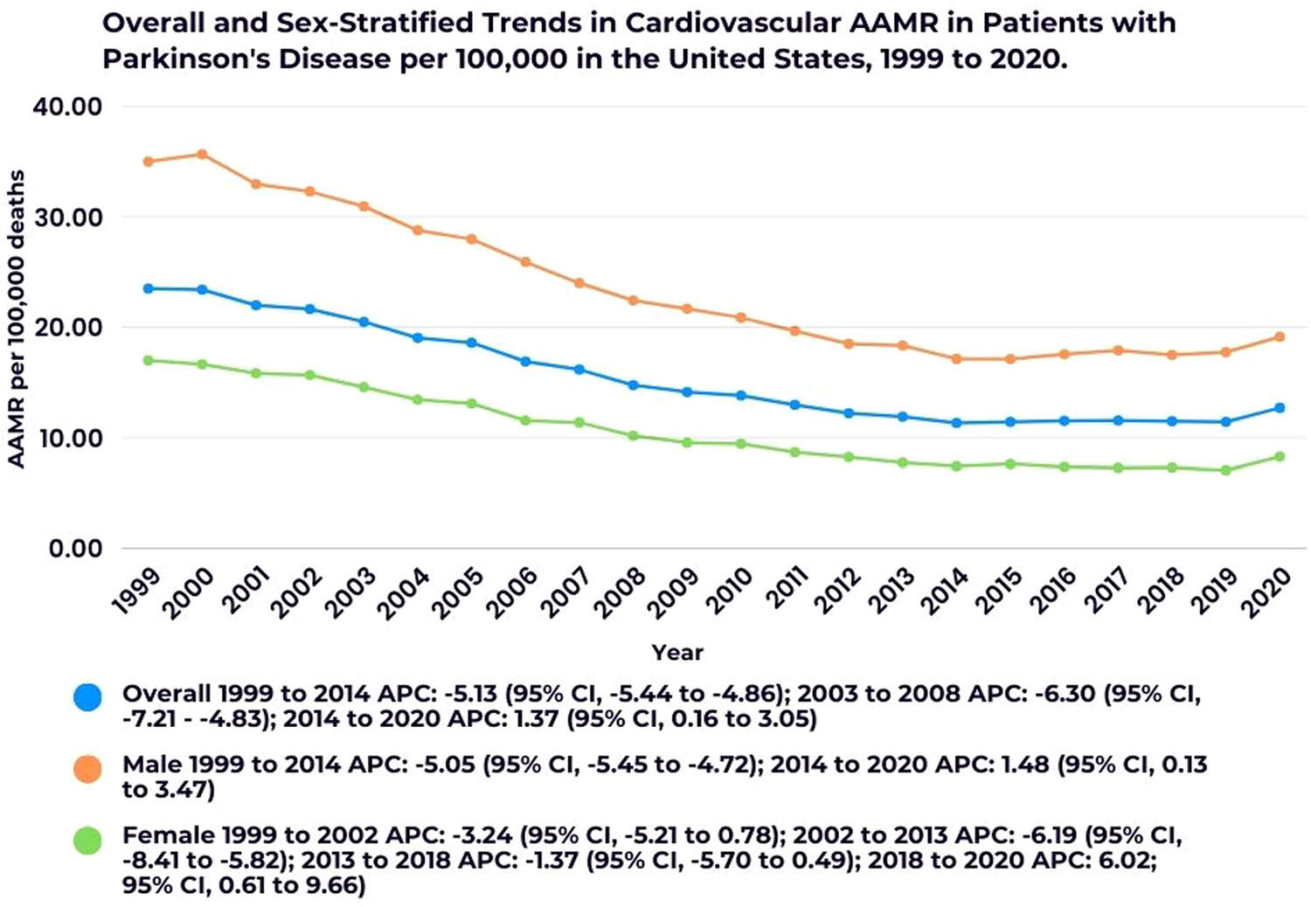
Overall and sex‐stratified trends in cardiovascular AAMR in patients with Parkinson's disease per 100 000 in the United States. * Indicates that the Annual Percent Change (APC) is significantly different from zero at the alpha = 0.05 level.

The AAMR for CVD with PD as a contributing factor declined from 23.48 per in 1999 to 12.69 per in 2020, with an AAPC of −3.32 (95% CI, −3.53 to −3.11). A more substantial decline was observed between 1999 and 2014 (APC: −5.13; 95% CI, −5.44 to −4.86), followed by a slight increase from 2014 to 2020 (APC: 1.37; 95% CI, 0.16 to 3.05. (Figure 1; Supporting Information S1: Table 2).

## 5 Gender‐Stratified Trends

Males consistently exhibited higher AAMRs than females across the study period. The overall AAMR for males was 22.58 (95% CI, 22.43–22.74), while females had an AAMR of 10.39 (95% CI, 10.30–10.47). Among males, the AAMR dropped significantly from 34.97 in 1999 to 17.12 in 2014 (APC: −5.05; 95% CI, −5.45 to −4.72), before rising slightly to 19.12 in 2020 (APC: 1.48; 95% CI, 0.13–3.47). In females, the AAMR initially decreased from 16.97 in 1999 to 15.65 in 2002 (APC: −3.24; 95% CI, −5.21 to 0.78), with a more pronounced decline to 7.74 in 2013 (APC: −6.19; 95% CI, −8.41 to −5.82). A slight additional decline occurred until 2018 (AAMR: 7.29; APC: −1.37; 95% CI, −5.70 to 0.49), followed by a significant increase from 2018 to 2020 (APC: 6.02; 95% CI, 0.61–9.66. (Figure 1; Supporting Information S1: Table 3).

## 6 Race‐Stratified Trends

**Figure 2:**
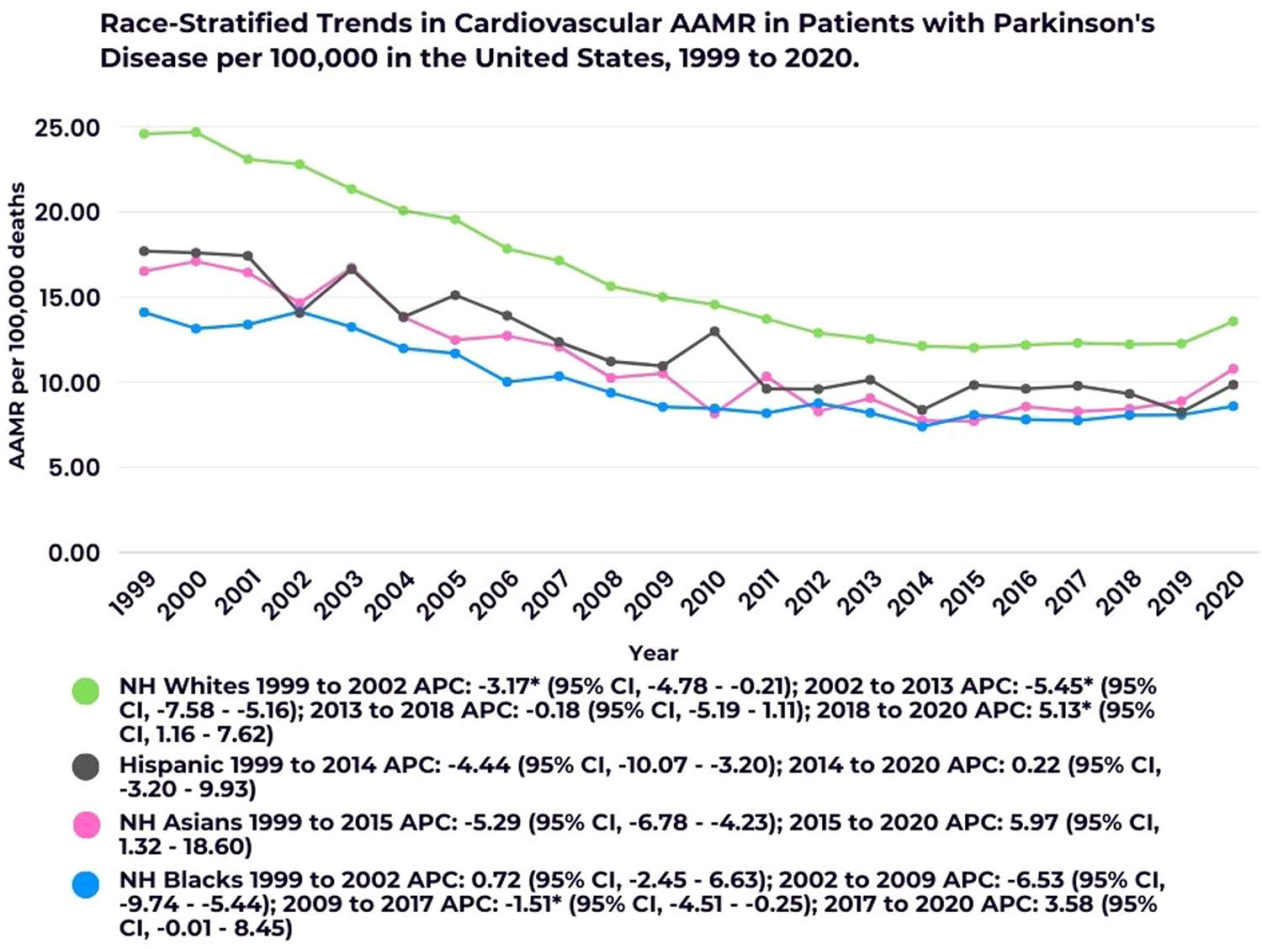
Race‐stratified trends in cardiovascular AAMR in patients with Parkinson's disease per 100 000 in the United States. * Indicates that the Annual Percent Change (APC) is significantly different from zero at the alpha = 0.05 level.

Race‐stratified trends revealed that NH White individuals had the highest AAMR, followed by Hispanic, NH Asian, and NH Black populations. The overall AAMR was 16.14 for NH Whites (95% CI, 16.05–16.23), 11.22 for Hispanics (95% CI, 10.94–11.50), 10.24 for NH Asians (95% CI, 9.88–10.60), and 9.65 for NH Blacks (95% CI, 9.42–9.88). NH Whites experienced a consistent decline from 1999 to 2018, with the steepest drop occurring between 2002 and 2013 (APC: −5.45; 95% CI, −7.58 to −5.16). However, a significant rise was observed from 2018 to 2020 (APC: 5.13; 95% CI, 1.16–7.62). In the Hispanic population, AAMRs declined steadily from 1999 to 2014 (APC: −4.44; 95% CI, −10.07 to −3.20) and remained stable until 2020 (APC: 0.22; 95% CI, −3.20 to 9.93). NH Asians exhibited a significant reduction from 1999 to 2015 (APC: −5.29; 95% CI, −6.78 to −4.23), followed by a notable increase from 2015 to 2020 (APC: 5.97; 95% CI, 1.32–18.60). For NH Blacks, the AAMR consistently declined from 1999 to 2017, with a marked reduction from 2002 to 2009 (APC: −6.53; 95% CI, −9.74 to −5.44). However, an upward trend emerged from 2017 to 2020 (APC: 3.58; 95% CI, 0.01–8.45) (Figure 2; Supporting Information S1: Table 4).

## 7 Urbanization‐Stratified Trends

**Figure 3:**
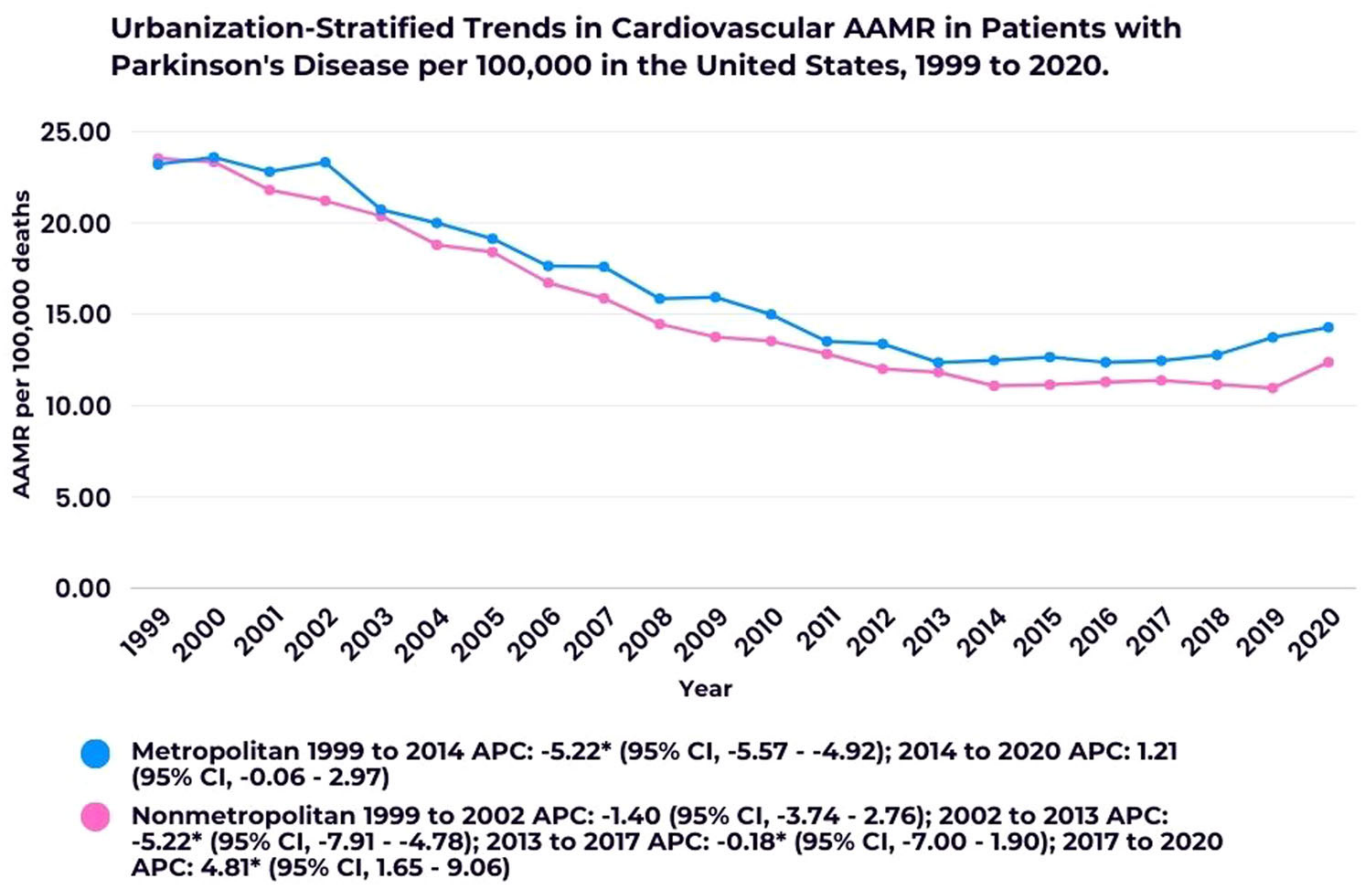
Urbanization‐stratified trends in cardiovascular AAMR in patients with Parkinson's disease per 100 000 in the United States. * Indicates that the Annual Percent Change (APC) is significantly different from zero at the alpha = 0.05 level.

Nonmetropolitan areas exhibited higher overall AAMRs (16.34; 95% CI, 16.14–16.54) compared to metropolitan areas (14.88; 95% CI, 14.80–14.97). In nonmetropolitan areas, the AAMR consistently decreased from 1999 to 2017, with a significant drop between 2002 and 2013 (APC: −5.22; 95% CI, −7.91 to −4.78). However, a sharp increase occurred from 2017 to 2020 (APC: 4.81; 95% CI, 1.65–9.06). Similarly, metropolitan areas showed a significant decline from 1999 to 2014 (APC: −5.22; 95% CI, −5.57 to −4.92), followed by a modest increase from 2014 to 2020 (APC: 1.21; 95% CI, −0.06 to 2.97) (Figure 3; Supporting Information S1: Table 5).

## 8 State‐Based Differences

**Figure 4:**
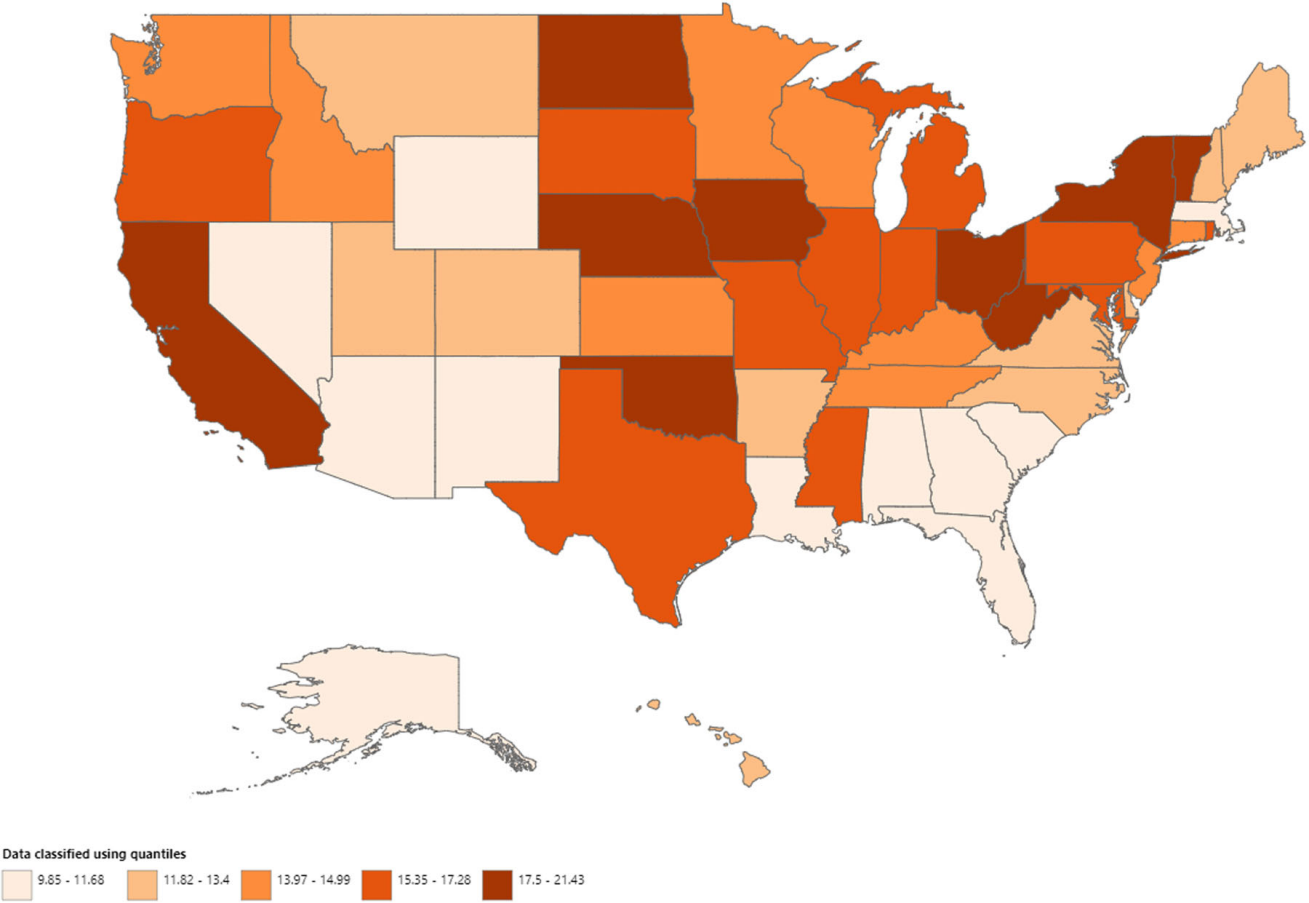
State‐wise map highlighting the states with highest mortality in the United States from 1999 to 2020.

Significant variation in AAMRs was observed across states, with the lowest rate in Georgia (9.85; 95% CI, 9.43–10.28) and the highest in Nebraska (21.43; 95% CI, 20.26–22.61). States in the highest 90th percentile, including Nebraska, California, Oklahoma, and Vermont, exhibited AAMRs approximately double those in the lowest 10th percentile, which included Georgia, Arizona, Nevada, and Massachusetts. The states with the highest proportion of total deaths were California (14.43%), New York (7.87%), and Texas (6.25%) (Figure 4; Supporting Information S1: Table 6).

## 9 Underlying Causes of Death

**Figure 5:**
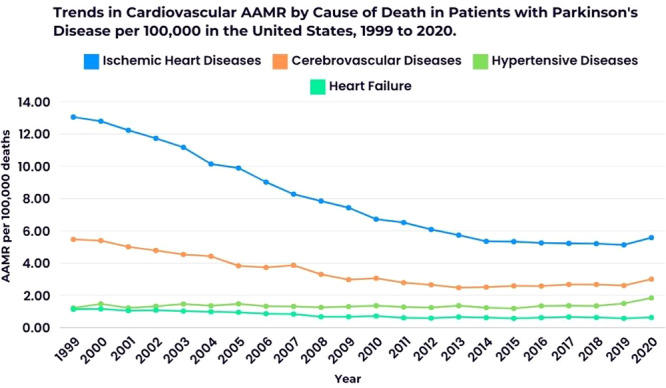
Trends in cardiovascular AAMR by cause of death in patients with Parkinson's disease per 100 000 in the United States. * Indicates that the Annual Percent Change (APC) is significantly different from zero at the alpha = 0.05 level.

Ischemic heart disease emerged as the most common underlying cause of death among decedents with PD, accounting for 50.71% of deaths, followed by cerebrovascular diseases (22.37%), hypertensive diseases (9.01%), and heart failure (5.11%). The AAMR decreased for all CVD‐related causes of death, except hypertensive diseases, which remained stable until 2018 before experiencing a significant rise from 2018 to 2020 (APC: 18.69; 95% CI, 4.04–25.37) (Figure 5).

## 10 Discussion

The examination of mortality trends from 1999 to 2020 for older adults with CVD and PD reveals significant insights. Results show a decline in the AAMR from 1999 to 2014, followed by a moderate increase from 2014 to 2020. This shift prompts questions about the underlying drivers of mortality in this group and highlights implications for public health planning.

The initial reduction in AAMR between 1999 and 2014 may be linked to medical advancements and heightened awareness of CVD and PD. New pharmacological developments, such as levodopa for PD, have shown substantial benefits for patient outcomes [18]. Additionally, improved control of cardiovascular risk factors like hypertension and hyperlipidemia likely contributed to reduced mortality rates during this time [19]. However, the rise in AAMR after 2014 suggests that these improvements may be plateauing, possibly due to population aging, an increase in comorbid conditions, and healthcare access disparities.

Gender differences are also evident, with males consistently showing higher AAMR than females across the study period, consistent with literature documenting higher rates of ischemic heart disease and other cardiovascular conditions in men [20, 21]. Variations in treatment practices by gender could contribute as well; for example, women with coronary artery disease are sometimes less likely to receive ideal treatment, potentially exacerbating their mortality risks [22]. Physiological factors, including hormonal variations, may also account for part of these differences [23].

Ethnic disparities in AAMR further underscore the findings, with White and Hispanic individuals experiencing the highest AAMR from 1999 to 2020. Broader social determinants, such as socioeconomic challenges, healthcare accessibility, and lifestyle factors, may disproportionately impact these groups [24]. Studies show that Hispanic populations, for example, often encounter barriers to healthcare, leading to delays in diagnosis and treatment for CVD and PD [25]. Additionally, overlapping gender and ethnic factors can intensify these disparities, indicating a need for targeted interventions for these populations [26].

The AAMR for CVD and Parkinson's disease were significantly elevated in rural areas compared to urban centers. A key reason for the higher mortality rates in nonmetropolitan regions is the socioeconomic disadvantage prevalent in these areas. Research indicates that rural residents often have lower educational attainment and income, both of which are crucial for health outcomes [27, 28]. The relationship between socioeconomic status and health literacy is particularly significant; lower health literacy can result in poor management of chronic conditions like CVD and PD [27, 29]. Additionally, rural populations generally exhibit a higher prevalence of risk factors such as obesity, hypertension, and smoking, further worsening mortality rates linked to these diseases [30]. Limited access to healthcare services, including a scarcity of medical facilities and specialists, can also delay diagnosis and treatment, contributing to poorer outcomes compared to urban populations [31, 32].

Disparities in AAMRs between states can be attributed to factors such as access to quality healthcare, socioeconomic conditions, and lifestyle choices. States with higher rates often have less healthcare access and more chronic illness. Public health initiatives, demographic differences, and environmental influences also play significant roles. Together, these elements contribute to the observed variations in mortality outcomes.

Our analysis also reveals that ischemic heart disease is a significant cause of death among individuals with PD, contributing to the overall mortality burden in this population. Existing literature indicates a notable prevalence of CVD among older populations, particularly those with neurodegenerative conditions like PD [33, 34]. While overall cardiovascular mortality has shown a decline, the burden of CVD remains high, contributing to increased mortality rates in older adults [33, 35]. Furthermore, the intersection of PD and CVD is critical, as older patients with PD often present with multiple comorbidities, which can exacerbate their cardiovascular risk [36]. This highlights the need for targeted interventions to manage cardiovascular health in this vulnerable population, as improvements in life expectancy may be undermined by the high mortality associated with ischemic heart disease [33, 34, 35].

The recent increase in AAMR across genders and ethnic groups raises questions about the effectiveness of current public health efforts. The limited progress in cardiovascular health, particularly for younger women, points to the need for more comprehensive strategies [25]. Potential solutions could include enhanced public awareness initiatives for early risk detection and management in high‐risk groups, along with efforts to ensure equitable healthcare access and treatment across gender and ethnic lines [37]. Moreover, incorporating exercise and lifestyle interventions in the management of both CVD and PD may yield significant benefits. Physical activity, shown to improve motor and non‐motor symptoms of PD, could enhance quality of life and reduce mortality risk [38]. Promoting regular physical activity among older adults, especially those with PD, could play a crucial role in a holistic strategy to mitigate mortality associated with these conditions.

### 10.1 Limitations

Our study is subject to several limitations that should be acknowledged. Primarily, the retrospective nature of the design hampers our capacity to ensure data quality and introduces potential biases inherent in the analysis of previously collected information. Additionally, our reliance on ICD codes may lead to misclassification or underreporting of CVD and PD as causes of death, with changes in coding practices over time further affecting trend consistency. The absence of clinical details—such as disease stage and treatment history—alongside unmeasured confounders like socioeconomic status and healthcare access, restricts the analytical depth of our findings. Furthermore, this study cannot establish causality, and the generalizability of the results may be limited by reporting biases and regional variations in healthcare quality.

## 11 Conclusion

In conclusion, this analysis of mortality trends in CVD and PD reveals a complex interplay of factors including gender, ethnicity, and healthcare access. While the initial decrease in AAMR is promising, the recent uptick highlights the ongoing need for targeted interventions. By addressing disparities in healthcare access, refining public health strategies, and incorporating lifestyle modifications, we may work toward reversing these mortality trends and improving health outcomes for older adults managing these chronic diseases.

## Author Contributions

Conceptualization: Muzamil Akhtar; Data curation: Muzamil Akhtar and Hanzala Ahmed Farooqi; Methodology: Rayyan Nabi; Supervision: Raheel Ahmed; Resources: Sabahat Ul Ain Munir Abbasi; Writing–Original Draft: Muzamil Akhtar, Hanzala Ahmed Farooqi, Sabahat Ul Ain Munir Abbasi and Rayyan Nabi; Writing–Review and editing: Raheel Ahmed and Sarah MacKenzie Picker.

## Ethics Statement

The authors have nothing to report.

## Conflicts of Interest

The authors declare no conflicts of interest.

## Supporting information

Supporting information.

## Data Availability

The data that support the findings of this study are available from the corresponding author upon reasonable request.

## References

[1] A. W. Willis , E. Roberts , J. C. Beck , et al., “Incidence of Parkinson Disease in North America,” NPJ Parkinson's Disease 8 (2022): 170. 10.1038/s41531-022-00410-y PMC9755252 36522332

[2] A. M. Arnold , B. M. Psaty , L. H. Kuller , et al., “Incidence of Cardiovascular Disease in Older Americans: The Cardiovascular Health Study,” Journal of the American Geriatrics Society 53 (2005): 211–218. 15673343 10.1111/j.1532-5415.2005.53105.x

[3] J. Potashkin , X. Huang , C. Becker , H. Chen , T. Foltynie , and C. Marras , “Understanding the Links Between Cardiovascular Disease and Parkinson's Disease,” Movement Disorders: Official Journal of the Movement Disorder Society 35 (2020): 55–74. 31483535 10.1002/mds.27836 PMC6981000

[4] A. Yalcin , V. Atmis , O. Karaarslan Cengiz , et al., “Evaluation of Cardiac Autonomic Functions in Older Parkinson's Disease Patients: A Cross‐Sectional Study,” Aging and Disease 7 (2016): 28. 26816661 10.14336/AD.2015.0819 PMC4723231

[5] D. C. Velseboer , R. J. de Haan , W. Wieling , D. S. Goldstein , and R. M. A. de Bie , “Prevalence of Orthostatic Hypotension in Parkinson's Disease: A Systematic Review and Meta‐Analysis,” Parkinsonism & Related Disorders 17 (2011): 724–729. 21571570 10.1016/j.parkreldis.2011.04.016 PMC5199613

[6] L. Grosu , A. Grosu , D. Crisan , A. Zlibut , and L. Perju‑Dumbrava , “Parkinson's Disease and Cardiovascular Involvement: Edifying Insights (Review),” Biomedical Reports 18 (2023): 25. 36846617 10.3892/br.2023.1607 PMC9944619

[7] R. Dhall and D. L. Kreitzman , “Advances in Levodopa Therapy for Parkinson Disease,” Neurology 86 (2016). 10.1212/WNL.0000000000002510 27044646

[8] C. Noack , C. Schroeder , K. Heusser , and A. Lipp , “Cardiovascular Effects of Levodopa in Parkinson's Disease,” Parkinsonism & Related Disorders 20 (2014): 815–818. 24819390 10.1016/j.parkreldis.2014.04.007

[9] Z. Y. Günaydın , F. F. Özer , A. Karagöz , et al., “Evaluation of Cardiovascular Risk in Patients With Parkinson Disease Under Levodopa Treatment,” Journal of Geriatric Cardiology: JGC 13 (2016): 75–80. 26918017 10.11909/j.issn.1671-5411.2016.01.003 PMC4753016

[10] J. Kho , A. Ioannou , A. K. J. Mandal , et al., “Long Term Use of Donepezil and QTc Prolongation,” Clinical Toxicology 59 (2021): 208–214. 32609550 10.1080/15563650.2020.1788054

[11] Y. Chen and G. W. Dorn , “PINK1‐Phosphorylated Mitofusin 2 Is a Parkin Receptor for Culling Damaged Mitochondria,” Science (1979) 340 (2013): 471–475. 10.1126/science.1231031 PMC3774525 23620051

[12] L. Cuenca‐Bermejo , P. Almela , J. Navarro‐Zaragoza , et al., “Cardiac Changes in Parkinson's Disease: Lessons From Clinical and Experimental Evidence,” International Journal of Molecular Sciences 22 (2021): 13488. 34948285 10.3390/ijms222413488 PMC8705692

[13] U. A. Mukherjee , S.‐B. Ong , S.‐G. Ong , and D. J. Hausenloy , “Parkinson's Disease Proteins: Novel Mitochondrial Targets for Cardioprotection,” Pharmacology & Therapeutics 156 (2015): 34–43. 26481155 10.1016/j.pharmthera.2015.10.005 PMC4667215

[14] B. S. Sokhal , S. Prasanna Kumar Menon , T. Shepherd , S. Muller , A. Arora , and C. Mallen , “Temporal Trends in Parkinson's Disease Related Mortality From 1999–2020: A National Analysis,” NIHR Open Research 4 (2024): 50. 39830305 10.3310/nihropenres.13623.1 PMC11739699

[15] M. H. Maqsood , K. M. Talha , A. M. K. Minhas , et al., “CDC‐WONDER Database Analysis of COVID‐19 and Cardiovascular Disease‐Related Mortality,” Journal of the American College of Cardiology 81 (2023): 1743–1745. 37100492 10.1016/j.jacc.2023.02.044 PMC10124577

[16] R. Aggarwal , N. Chiu , E. C. Loccoh , D. S. Kazi , R. W. Yeh , and R. K. Wadhera , “Rural‐Urban Disparities,” Journal of the American College of Cardiology 77 (2021): 1480–1481. 33736831 10.1016/j.jacc.2021.01.032 PMC8210746

[17] D. D. Ingram and S. J. Franco , “2013 NCHS Urban‐Rural Classification Scheme for Counties,” Vital and Health Statistics. Series 2, Data Evaluation and Methods Research 2 (2014): 1–73. 24776070

[18] R. J. Marttila and U. K. Rinne , “Changing Epidemiology of Parkinson's Disease: Predicted Effects of Levodopa Treatment,” Acta Neurologica Scandinavica 59 (2009): 80–87. 10.1111/j.1600-0404.1979.tb02914.x 452843

[19] L. Mosca , E. Barrett‐Connor , and N. Kass Wenger , “Sex/Gender Differences in Cardiovascular Disease Prevention,” Circulation 124 (2011): 2145–2154. 22064958 10.1161/CIRCULATIONAHA.110.968792 PMC3362050

[20] K. Silander , M. Alanne , K. Kristiansson , et al., “Gender Differences in Genetic Risk Profiles for Cardiovascular Disease,” PLoS One 3 (2008): e3615. 18974842 10.1371/journal.pone.0003615 PMC2574036

[21] U. Alehagen and D. Wagsater , “Gender Difference and Genetic Variance in Lipoprotein Receptor‐Related Protein 1 Is Associated With Mortality,” Biomedical Reports (2019): 3–10. 10.3892/br.2019.1217 PMC6566454 31258899

[22] A. G. Vouyouka , N. N. Egorova , A. Salloum , et al., “Lessons Learned from the Analysis of Gender Effect on Risk Factors and Procedural Outcomes of Lower Extremity Arterial Disease,” Journal of Vascular Surgery 52 (2010): 1196–1202. 20674247 10.1016/j.jvs.2010.05.106

[23] M. E. Mendelsohn and R. H. Karas , “Molecular and Cellular Basis of Cardiovascular Gender Differences,” Science 308 (2005): 1583–1587. 15947175 10.1126/science.1112062

[24] T. Sallam and K. E. Watson , “Predictors of Cardiovascular Risk in Women,” Women's Health 9 (2013): 491–498. 10.2217/whe.13.44 PMC6097244 24007254

[25] M. Garcia , S. L. Mulvagh , C. N. Bairey Merz , J. E. Buring , and J. E. Manson , “Cardiovascular Disease in Women,” Circulation Research 118 (2016): 1273–1293. 27081110 10.1161/CIRCRESAHA.116.307547 PMC4834856

[26] P. Russo , A. Siani , M. A. Miller , et al., “Genetic Variants of Y Chromosome Are Associated With a Protective Lipid Profile in Black Men,” Arteriosclerosis, Thrombosis, and Vascular Biology 28 (2008): 1569–1574. 18511697 10.1161/ATVBAHA.108.168641

[27] L. Alston , K. L. Peterson , J. P. Jacobs , S. Allender , and M. Nichols , “Quantifying the Role of Modifiable Risk Factors in the Differences in Cardiovascular Disease Mortality Rates Between Metropolitan and Rural Populations in Australia: A Macrosimulation Modelling Study,” BMJ Open 7 (2017): e018307. 10.1136/bmjopen-2017-018307 PMC5695309 29101149

[28] G. Singh , “Widening Geographical Disparities in Cardiovascular Disease Mortality in the United States, 1969–2011,” International Journal of MCH and AIDS (IJMA) 3 (2014): 134–149. PMC5005988 27621993

[29] H. G. Peach and N. E. Bath , “Prevalence and Sociodemographic Determinants of Cardiovascular Risk in a Rural Area,” Australian Journal of Rural Health 7 (1999): 23–27. 10373812 10.1046/j.1440-1584.1999.00198.x

[30] R. Disler , K. Glenister , and J. Wright , “Rural Chronic Disease Research Patterns in the United Kingdom, United States, Canada, Australia and New Zealand: A Systematic Integrative Review,” BMC Public Health 20 (2020): 770. 32448173 10.1186/s12889-020-08912-1 PMC7247224

[31] H. D. Vu , “Mortality After Acute Myocardial Infarction Is Lower in Metropolitan Regions Than in Non‐Metropolitan Regions,” Journal of Epidemiology & Community Health 54 (2000): 590–595. 10890870 10.1136/jech.54.8.590 PMC1731723

[32] J. Cossman , W. James , and J. K. Wolf , “The Differential Effects of Rural Health Care Access on Race‐Specific Mortality,” SSM ‐ Population Health 3 (2017): 618–623. 29349249 10.1016/j.ssmph.2017.07.013 PMC5769119

[33] P. Lloyd‐Sherlock , S. Ebrahim , R. Martinez , M. McKee , and P. Ordunez , “Reducing the Cardiovascular Disease Burden for People of All Ages in the Americas Region: Analysis of Mortality Data, 2000–15,” Lancet Global Health 7 (2019): e604–e612. 31000130 10.1016/S2214-109X(19)30069-5

[34] P. Ordunez , E. Prieto‐Lara , V. Pinheiro Gawryszewski , A. J. M. Hennis , and R. S. Cooper , “Premature Mortality From Cardiovascular Disease in the Americas – Will the Goal of a Decline of ‘25% by 2025’ Be Met?,” PLoS One 10 (2015): e0141685. 26512989 10.1371/journal.pone.0141685 PMC4626103

[35] I. Akushevich , J. Kravchenko , S. Ukraintseva , K. Arbeev , and A. I. Yashin , “Time Trends of Incidence of Age‐Associated Diseases in the Us Elderly Population: Medicare‐Based Analysis,” Age and Ageing 42 (2013): 494–500. 23482353 10.1093/ageing/aft032 PMC3684110

[36] C. Connell , R. Gellatly , M. Dooley , and J. Shaw , “A Case of St Elevation Myocardial Infarction Precipitated by Methylphenidate Therapy for Gait Freeze,” Journal of Pharmacy Practice and Research 45 (2015): 174–177.

[37] N. Wenger , “Tailoring Cardiovascular Risk Assessment and Prevention for Women: One Size Does Not Fit All,” Global Cardiology Science and Practice 2017 (2017). 10.21542/gcsp.2017.1 PMC5621718 28971101

[38] M. Fayyaz , S. S. Jaffery , F. Anwar , A. Zil‐E‐Ali , and I. Anjum , “The Effect of Physical Activity in Parkinson's Disease: A Mini‐Review,” Cureus (2018). 10.7759/cureus.2995 PMC6143369 30245949

