# Correction to “Trends in Mortality Due to Cardiovascular Diseases Among Patients With Parkinson's Disease in the United States: A Retrospective Analysis”

**DOI:** 10.1002/clc.70168

**Published:** 2025-06-24

Akhtar M, Farooqi HA, Nabi R, et al. Trends in mortality due to cardiovascular diseases among patients with Parkinson's disease in the United States: a retrospective analysis. *Clinical Cardiology* 48, no. 1 (2025): e70079. doi:10.1002/clc.70079

The *y*‐axis label of Figures 1–3 and 5 should read “AAMR per 100,000 individuals” (not “AAMR per 100,000 deaths”). Additionally, in the color key section of Figure 1, the text next to the blue circle should state: “Overall 1999 to 2003 APC: −5.13 (95% CI: −5.44 to −4.86); 2003 to 2014 APC: −6.30”. The corrected figures appear below.

**Figure:**
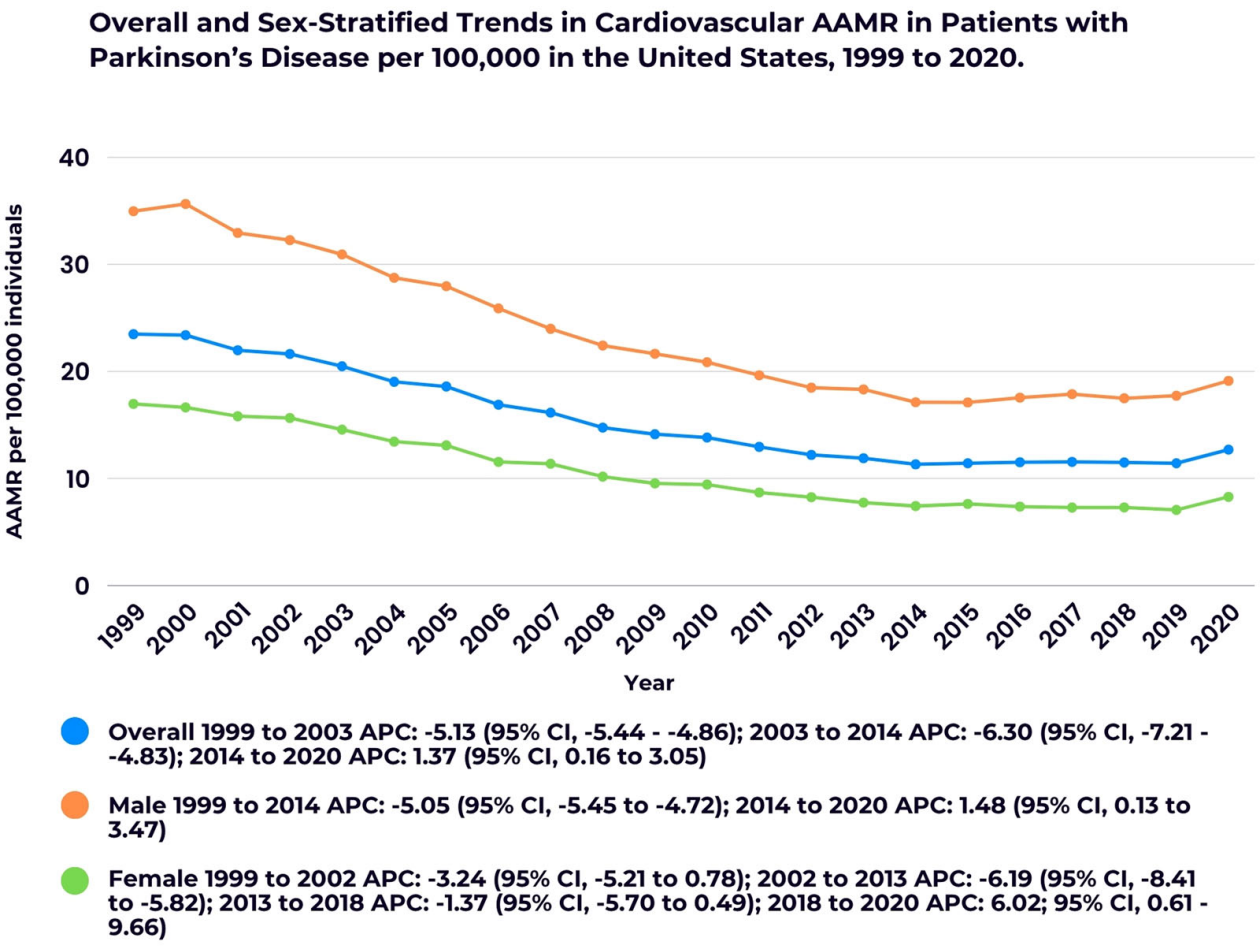
FIGURE 1

**Figure:**
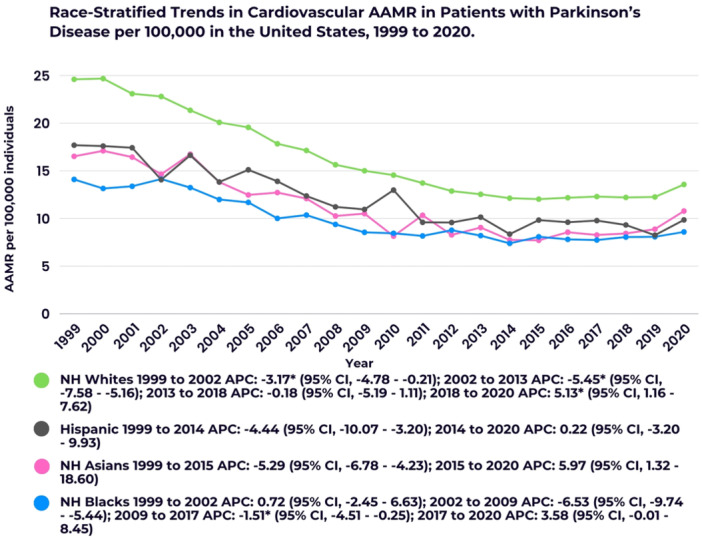
FIGURE 2

**Figure:**
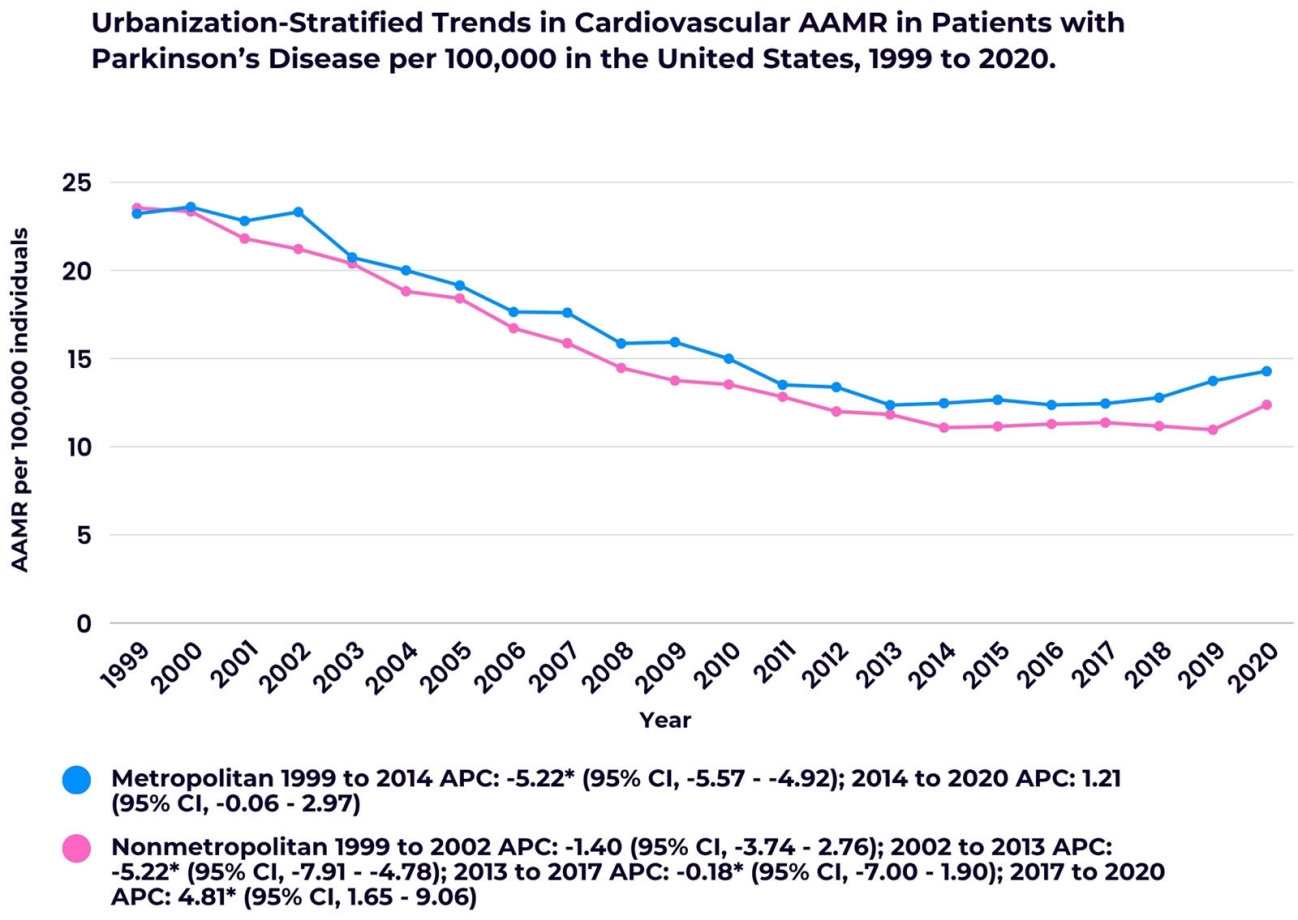
FIGURE 3

**Figure:**
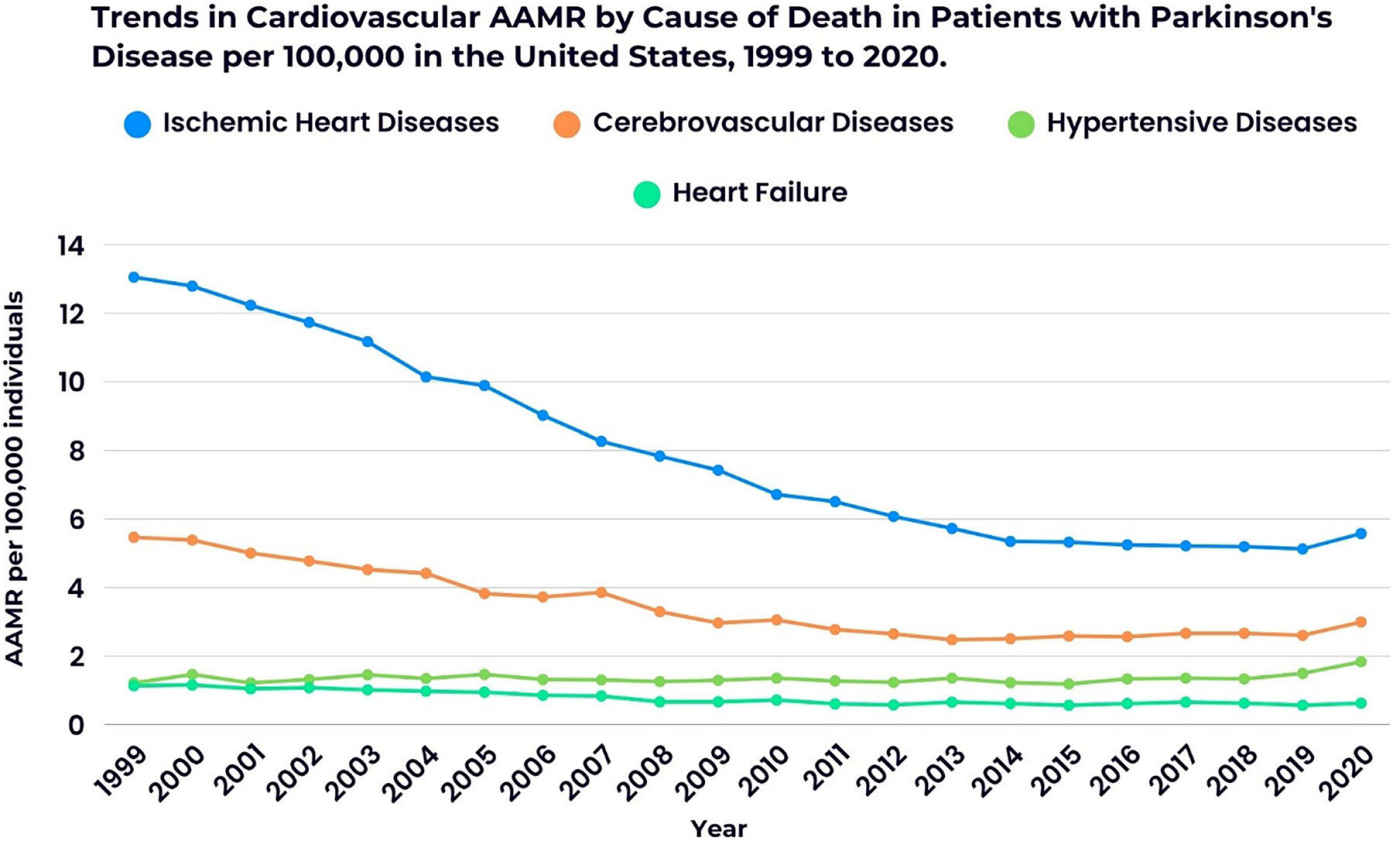
FIGURE 5

The authors would like to clarify that these revisions are typographical oversights and do not affect the integrity of their findings.

