# Supplementary material for: Trends in Mortality Due to Cardiovascular Diseases Among Patients With Parkinson's Disease in the United States: A Retrospective Analysis
Source: Clin Cardiol. 2025 Jan 16;48(1):e70079. doi: 10.1002/clc.70079 (PMC11736633; doi:10.1002/clc.70079)
Supplement: Supplementary file 1 — Supporting information. [file CLC-48-e70079-s001.docx]

**Supplementary File:**

| **Table 1:** Frequency and age adjusted mortality rates per 100,000 deaths in adults aged 65+ in the United States, 1999 to 2020 | | | |
| --- | --- | --- | --- |
|  | **Deaths** | **Population** | **Overall AAMR per 100,000 deaths (95% CI)** |
| Entire Cohort | 138151 | 928476665 | 15.11 (15.03 - 15.19) |
| ***Sex*** |  |  |  |
| Male | 78917 | 400844539 | 22.58 (22.43 - 22.74) |
| Female | 59234 | 527632126 | 10.39 (10.3 - 10.47) |
| **Race** |  |  |  |
| NH Asian or Pacific Islander | 3131 | 35222833 | 10.24 (9.88 -10.6) |
| NH Black or African American | 6992 | 80673683 | 9.65 (9.42 -9.88) |
| NH White | 121052 | 742709179 | 16.14 (16.05 -16.23) |
| NH American Indian or Alaska Native | 371 | 4746248 | 9.7 (8.7 -10.7) |
| Hispanic | 6332 | 65124722 | 11.22 (10.94 -11.5) |
| **Urbanization** |  |  |  |
| Metropolitan | 111351 | 760496648 | 16.88 (16.8 – 16.97) |
| Nonmetropolitan | 26800 | 167978547 | 16.34 (16.14 – 16.54) |
| **Underlying Cause of Death** |  |  |  |
| Ischemic Heart Diseases | 70065 | 928476665 | 7.65 (7.59 - 7.7) |
| Cerebrovascular Diseases | 30907 | 928476665 | 3.41 (3.37 - 3.45) |
| Hypertensive Diseases | 12458 | 928476665 | 1.35 (1.33 - 1.37) |
| Heart Failure | 7062 | 928476665 | 0.78 (0.76 - 0.79) |

**Supplementary Table 1:** Frequency and age adjusted mortality rates per 100,000 deaths in adults aged 65+ in the United States, 1999 to 2020

| **Table 2:** Annual age adjusted mortality rates per 100,000 deaths in adults aged 65+ in the United States, 1999 to 2020 | |
| --- | --- |
| **Year** | **Age Adjusted Mortality Rate per 100,000 deaths (95% CI)** |
| 1999 | 23.48 (22.96 - 23.99) |
| 2000 | 23.39 (22.88 - 23.89) |
| 2001 | 21.98 (21.49 - 22.47) |
| 2002 | 21.63 (21.15 - 22.11) |
| 2003 | 20.48 (20.01 - 20.94) |
| 2004 | 19.03 (18.59 - 19.48) |
| 2005 | 18.59 (18.15 - 19.03) |
| 2006 | 16.89 (16.48 - 17.31) |
| 2007 | 16.15 (15.75 - 16.55) |
| 2008 | 14.75 (14.37 - 15.13) |
| 2009 | 14.13 (13.76 - 14.5) |
| 2010 | 13.82 (13.46 - 14.19) |
| 2011 | 12.95 (12.6 - 13.29) |
| 2012 | 12.2 (11.87 - 12.53) |
| 2013 | 11.89 (11.56 - 12.21) |
| 2014 | 11.32 (11.01 - 11.64) |
| 2015 | 11.42 (11.11 - 11.73) |
| 2016 | 11.51 (11.2 - 11.82) |
| 2017 | 11.55 (11.24 - 11.86) |
| 2018 | 11.49 (11.19 - 11.79) |
| 2019 | 11.42 (11.12 - 11.72) |
| 2020 | 12.69 (12.38 - 13) |

**Supplementary Table 2:** Annual age adjusted mortality rates per 100,000 deaths in adults aged 65+ in the United States, 1999 to 2020

| **Table 3:** Age adjusted mortality rates per 100,000 deaths stratified by sex in adults aged 65+ in the United States, 1999 to 2020 | | |
| --- | --- | --- |
|  | **Age Adjusted Mortality Rate per 100,000 deaths (95% CI)** | |
| **Year** | **Female** | **Male** |
| 1999 | 16.97 (16.43 - 17.51) | 34.97 (33.9 - 36.04) |
| 2000 | 16.64 (16.1 - 17.17) | 35.65 (34.58 - 36.72) |
| 2001 | 15.81 (15.29 - 16.33) | 32.94 (31.92 - 33.95) |
| 2002 | 15.65 (15.14 - 16.17) | 32.27 (31.27 - 33.27) |
| 2003 | 14.56 (14.07 - 15.05) | 30.93 (29.96 - 31.89) |
| 2004 | 13.44 (12.96 - 13.91) | 28.75 (27.83 - 29.67) |
| 2005 | 13.09 (12.63 - 13.55) | 27.96 (27.06 - 28.85) |
| 2006 | 11.55 (11.12 - 11.98) | 25.89 (25.04 - 26.74) |
| 2007 | 11.37 (10.94 - 11.79) | 23.98 (23.18 - 24.79) |
| 2008 | 10.17 (9.77 - 10.57) | 22.42 (21.66 - 23.19) |
| 2009 | 9.54 (9.15 - 9.93) | 21.65 (20.91 - 22.39) |
| 2010 | 9.43 (9.05 - 9.82) | 20.86 (20.14 - 21.59) |
| 2011 | 8.69 (8.33 - 9.05) | 19.64 (18.95 - 20.32) |
| 2012 | 8.25 (7.9 - 8.6) | 18.48 (17.82 - 19.13) |
| 2013 | 7.74 (7.4 - 8.07) | 18.32 (17.68 - 18.97) |
| 2014 | 7.42 (7.1 - 7.75) | 17.12 (16.5 - 17.73) |
| 2015 | 7.62 (7.29 - 7.95) | 17.1 (16.5 - 17.71) |
| 2016 | 7.37 (7.05 - 7.69) | 17.55 (16.95 - 18.16) |
| 2017 | 7.28 (6.97 - 7.6) | 17.88 (17.28 - 18.48) |
| 2018 | 7.29 (6.98 - 7.6) | 17.49 (16.9 - 18.07) |
| 2019 | 7.06 (6.76 - 7.36) | 17.73 (17.15 - 18.31) |
| 2020 | 8.28 (7.96 - 8.61) | 19.12 (18.52 - 19.71) |

**Supplementary Table 3:** Age adjusted mortality rates per 100,000 deaths stratified by sex in adults aged 65+ in the United States, 1999 to 2020

| **Table 4:** Age adjusted mortality rates per 100,000 deaths stratified by race in adults aged 65+ in the United States, 1999 to 2020 | | | | |
| --- | --- | --- | --- | --- |
|  | **Age Adjusted Mortality Rate per 100,000 deaths (95% CI)** | | | |
| **Year** | **NH Asian or Pacific Islander** | **Black or African American** | **NH White** | **Hispanic or Latino** |
| 1999 | 16.52 (13.24 -19.8) | 14.1 (12.66 -15.55) | 24.6 (24.04 -25.17) | 17.69 (15.39 -19.98) |
| 2000 | 17.1 (13.91 -20.3) | 13.15 (11.76 -14.54) | 24.68 (24.12 -25.24) | 17.6 (15.39 -19.81) |
| 2001 | 16.44 (13.46 -19.42) | 13.38 (11.99 -14.78) | 23.09 (22.54 -23.63) | 17.42 (15.29 -19.54) |
| 2002 | 14.64 (11.92 -17.37) | 14.14 (12.71 -15.57) | 22.8 (22.26 -23.33) | 14.07 (12.18 -15.95) |
| 2003 | 16.74 (13.93 -19.55) | 13.24 (11.86 -14.61) | 21.35 (20.83 -21.86) | 16.63 (14.62 -18.64) |
| 2004 | 13.82 (11.33 -16.31) | 11.99 (10.69 -13.29) | 20.08 (19.59 -20.58) | 13.83 (12.07 -15.59) |
| 2005 | 12.47 (10.19 -14.76) | 11.69 (10.42 -12.96) | 19.56 (19.08 -20.05) | 15.11 (13.32 -16.9) |
| 2006 | 12.72 (10.47 -14.97) | 10.01 (8.85 -11.18) | 17.84 (17.38 -18.3) | 13.9 (12.23 -15.57) |
| 2007 | 12.09 (9.99 -14.2) | 10.36 (9.18 -11.53) | 17.14 (16.69 -17.59) | 12.36 (10.83 -13.89) |
| 2008 | 10.26 (8.39 -12.12) | 9.38 (8.28 -10.47) | 15.64 (15.21 -16.07) | 11.22 (9.79 -12.64) |
| 2009 | 10.51 (8.67 -12.35) | 8.55 (7.51 -9.58) | 15.01 (14.6 -15.43) | 10.95 (9.59 -12.31) |
| 2010 | 8.15 (6.57 -9.72) | 8.45 (7.43 -9.47) | 14.55 (14.15 -14.96) | 12.98 (11.53 -14.43) |
| 2011 | 10.34 (8.65 -12.04) | 8.17 (7.19 -9.16) | 13.72 (13.32 -14.11) | 9.6 (8.41 -10.79) |
| 2012 | 8.28 (6.82 -9.75) | 8.77 (7.76 -9.77) | 12.88 (12.5 -13.26) | 9.58 (8.42 -10.74) |
| 2013 | 9.05 (7.57 -10.53) | 8.2 (7.25 -9.15) | 12.54 (12.17 -12.91) | 10.14 (8.98 -11.29) |
| 2014 | 7.75 (6.44 -9.07) | 7.39 (6.5 -8.28) | 12.13 (11.77 -12.49) | 8.36 (7.34 -9.38) |
| 2015 | 7.71 (6.45 -8.98) | 8.08 (7.17 -9) | 12.03 (11.68 -12.39) | 9.83 (8.76 -10.9) |
| 2016 | 8.56 (7.26 -9.87) | 7.81 (6.92 -8.7) | 12.18 (11.82 -12.53) | 9.61 (8.58 -10.63) |
| 2017 | 8.27 (7.03 -9.51) | 7.74 (6.87 -8.6) | 12.3 (11.94 -12.65) | 9.78 (8.77 -10.8) |
| 2018 | 8.43 (7.21 -9.64) | 8.05 (7.18 -8.92) | 12.21 (11.86 -12.56) | 9.32 (8.36 -10.28) |
| 2019 | 8.88 (7.68 -10.09) | 8.08 (7.23 -8.93) | 12.26 (11.91 -12.61) | 8.25 (7.36 -9.14) |
| 2020 | 10.78 (9.48 -12.08) | 8.59 (7.72 -9.45) | 13.57 (13.2 -13.93) | 9.85 (8.91 -10.8) |

**Supplementary Table 4:** Age adjusted mortality rates per 100,000 deaths stratified by race in adults aged 65+ in the United States, 1999 to 2020

| **Table 5:** Age adjusted mortality rates per 100,000 deaths stratified by urbanization in adults aged 65+ in the United States, 1999 to 2020 | | |
| --- | --- | --- |
|  | **Age Adjusted Mortality Rate per 100,000 deaths (95% CI)** | |
| **Year** | **Metropolitan** | **Nonmetropolitan** |
| 1999 | 23.53 (22.96 - 24.1) | 23.21 (22.05 - 24.36) |
| 2000 | 23.34 (22.78 - 23.91) | 23.59 (22.44 - 24.75) |
| 2001 | 21.8 (21.26 - 22.34) | 22.8 (21.67 - 23.94) |
| 2002 | 21.21 (20.68 - 21.74) | 23.31 (22.16 - 24.46) |
| 2003 | 20.38 (19.86 - 20.89) | 20.73 (19.65 - 21.81) |
| 2004 | 18.81 (18.32 - 19.3) | 20 (18.94 - 21.05) |
| 2005 | 18.41 (17.93 - 18.89) | 19.14 (18.11 - 20.16) |
| 2006 | 16.72 (16.27 - 17.18) | 17.64 (16.66 - 18.62) |
| 2007 | 15.87 (15.43 - 16.31) | 17.6 (16.63 - 18.58) |
| 2008 | 14.47 (14.06 - 14.89) | 15.85 (14.93 - 16.78) |
| 2009 | 13.75 (13.35 - 14.15) | 15.93 (15.01 - 16.84) |
| 2010 | 13.53 (13.13 - 13.92) | 14.99 (14.1 - 15.88) |
| 2011 | 12.83 (12.45 - 13.21) | 13.51 (12.68 - 14.35) |
| 2012 | 12 (11.64 - 12.37) | 13.38 (12.56 - 14.21) |
| 2013 | 11.83 (11.47 - 12.18) | 12.36 (11.57 - 13.15) |
| 2014 | 11.08 (10.74 - 11.43) | 12.47 (11.68 - 13.26) |
| 2015 | 11.15 (10.81 - 11.49) | 12.66 (11.88 - 13.45) |
| 2016 | 11.29 (10.96 - 11.63) | 12.37 (11.59 - 13.14) |
| 2017 | 11.37 (11.03 - 11.7) | 12.45 (11.69 - 13.22) |
| 2018 | 11.17 (10.84 - 11.5) | 12.78 (12.01 - 13.55) |
| 2019 | 10.96 (10.64 - 11.28) | 13.73 (12.94 - 14.52) |
| 2020 | 12.38 (12.05 - 12.72) | 14.28 (13.48 - 15.07) |

**Supplementary Table 5:** Age adjusted mortality rates per 100,000 deaths stratified by urbanization in adults aged 65+ in the United States, 1999 to 2020

| **Table 6:** State wise age adjusted mortality rates per 100,000 deaths in adults aged 65+ in the United States, 1999 to 2020 | | |
| --- | --- | --- |
| **State** | **AAMR per 100,000 deaths (95% CI)** | **Percentage of Deaths** |
| Alabama | 10.92 (10.37 - 11.47) | 1.10% |
| Alaska | 10.35 (8.33 - 12.38) | 0.08% |
| Arizona | 10.08 (9.62 - 10.53) | 1.39% |
| Arkansas | 12.92 (12.18 - 13.66) | 0.85% |
| California | 20.38 (20.1 - 20.66) | 14.43% |
| Colorado | 13.4 (12.74 - 14.06) | 1.15% |
| Connecticut | 14.17 (13.5 - 14.84) | 1.28% |
| Delaware | 12.68 (11.35 - 14.01) | 0.25% |
| District of Columbia | 14.69 (12.81 - 16.56) | 0.17% |
| Florida | 10.34 (10.11 - 10.57) | 5.70% |
| Georgia | 9.85 (9.43 - 10.28) | 1.53% |
| Hawaii | 11.82 (10.82 - 12.81) | 0.40% |
| Idaho | 14.02 (12.89 - 15.15) | 0.43% |
| Illinois | 15.38 (14.98 - 15.78) | 4.18% |
| Indiana | 16.73 (16.15 - 17.31) | 2.29% |
| Iowa | 17.5 (16.72 - 18.28) | 1.43% |
| Kansas | 14.89 (14.1 - 15.69) | 0.99% |
| Kentucky | 14.86 (14.18 - 15.54) | 1.32% |
| Louisiana | 10.26 (9.69 - 10.83) | 0.91% |
| Maine | 12.73 (11.72 - 13.75) | 0.44% |
| Maryland | 17.28 (16.63 - 17.92) | 1.98% |
| Massachusetts | 10.16 (9.73 - 10.58) | 1.62% |
| Michigan | 16.35 (15.9 - 16.8) | 3.65% |
| Minnesota | 13.97 (13.4 - 14.55) | 1.67% |
| Mississippi | 16.76 (15.87 - 17.65) | 0.99% |
| Missouri | 16.23 (15.65 - 16.8) | 2.24% |
| Montana | 12.02 (10.83 - 13.21) | 0.29% |
| Nebraska | 21.43 (20.26 - 22.61) | 0.94% |
| Nevada | 10.12 (9.33 - 10.92) | 0.46% |
| New Hampshire | 13.14 (12.01 - 14.27) | 0.38% |
| New Jersey | 14.89 (14.43 - 15.34) | 3.02% |
| New Mexico | 11.64 (10.76 - 12.53) | 0.48% |
| New York | 17.55 (17.22 - 17.88) | 7.87% |
| North Carolina | 13.05 (12.61 - 13.49) | 2.47% |
| North Dakota | 17.66 (15.99 - 19.32) | 0.32% |
| Ohio | 18.65 (18.21 - 19.09) | 5.02% |
| Oklahoma | 20.24 (19.4 - 21.08) | 1.62% |
| Oregon | 15.35 (14.65 - 16.05) | 1.36% |
| Pennsylvania | 16.48 (16.12 - 16.85) | 5.66% |
| Rhode Island | 17.28 (15.95 - 18.6) | 0.48% |
| South Carolina | 11.68 (11.09 - 12.27) | 1.11% |
| South Dakota | 16.16 (14.68 - 17.63) | 0.34% |
| Tennessee | 14.99 (14.42 - 15.56) | 1.95% |
| Texas | 15.5 (15.17 - 15.83) | 6.25% |
| Utah | 13.11 (12.14 - 14.08) | 0.51% |
| Vermont | 19.27 (17.36 - 21.18) | 0.29% |
| Virginia | 12.04 (11.57 - 12.51) | 1.83% |
| Washington | 14.88 (14.32 - 15.43) | 2.00% |
| West Virginia | 17.5 (16.47 - 18.52) | 0.81% |
| Wisconsin | 14.35 (13.8 - 14.9) | 1.91% |
| Wyoming | 11.27 (9.56 - 12.97) | 0.12% |
| Alabama | 10.92 (10.37 - 11.47) | 1.10% |

**Supplementary Table 6:** State wise age adjusted mortality rates per 100,000 deaths in adults aged 65+ in the United States, 1999 to 2020
